# External characteristic determination of eggs and cracked eggs identification using spectral signature

**DOI:** 10.1038/srep21130

**Published:** 2016-02-17

**Authors:** Chuanqi Xie, Yong He

**Affiliations:** 1 College of Biosystems Engineering and Food Science, Zhejiang University, Hangzhou 310058, China

## Abstract

This study was carried out to use hyperspectral imaging technique for determining color (L*, a* and b*) and eggshell strength and identifying cracked chicken eggs. Partial least squares (PLS) models based on full and selected wavelengths suggested by regression coefficient (RC) method were established to predict the four parameters, respectively. Partial least squares-discriminant analysis (PLS-DA) and RC-partial least squares-discriminant analysis (RC-PLS-DA) models were applied to identify cracked eggs. PLS models performed well with the correlation coefficient (*r*_*p*_) of 0.788 for L*, 0.810 for a*, 0.766 for b* and 0.835 for eggshell strength. RC-PLS models also obtained the *r*_*p*_ of 0.771 for L*, 0.806 for a*, 0.767 for b* and 0.841 for eggshell strength. The classification results were 97.06% in PLS-DA model and 88.24% in RC-PLS-DA model. It demonstrated that hyperspectral imaging technique has the potential to be used to detect color and eggshell strength values and identify cracked chicken eggs.

Egg is one of the most important foods because of its healthy function to people. It is rich in protein, lipid and carbohydrates, and it also contains mineral elements and vitamins^1^. In food industry, eggs can be used for making cakes, breads, ice creams, etc. Also, eggs have therapeutic and diagnostic functions^2^. However, eggs can often be damaged due to their fragile eggshells. According to a previous study, cracked eggs made up from 6% to 8% of the total eggs production^3^. The eggshell is a natural protection for egg, and thus it is significant to get a high value of eggshell strength^4^. The eggshell strength, reflecting the resistance ability to damage, can protect eggs when they are in collecting, packaging, storage and transportation. It can be found the higher the eggshell strength, the stronger resist to damage. Cracked eggs can finally cause economic loss in two ways, one is that they cannot be sold at a high price, another is cracked eggs may raise the risk of bacterial contamination to intact eggs, which can even produce food quality and safety problems^5^,^6^,^7^. Therefore, cracked eggs should be identified and taken out before they are sent to the market. Egg color is also an important factor in egg industry. It can affect people’s choice due to the regional or national cultural preferences for different colors, directly affecting eggs’ production^8^,^9^. Thus, the determination of egg color and eggshell strength is of importance.

Hyperspectral imaging technique can produce spectral information as well as spatial information for objectives at the same time. A spatial hyperspectral cube can be generated when one sample was scanned by the hyperspectral imaging camera. The hyperspectral cube (hyperspectral image) contains a series of images covering the whole wavelengths, and each pixel for one image has both spectral and spatial information^10^. Because of this feature, it can be used to detect external characteristics, such as fruit defect^11^,^12^, color^13^ and sugar beet disease^14^, and internal information, such as moisture content^10^ and other chemical indexes^15^,^16^. By studying spectral features at different wavelengths, it may be possible for color and eggshell strength determination and cracked eggs identification, which can be seen in many previous studies. Wu *et al*. predicted beef color (L*, a* and b*) using hyperspectral imaging technique^17^. Wu *et al*. investigated color distribution in salmon fillet by using hyperspectral imaging^18^. Iqbal *et al*. detected the color in turkey hams by hyperspectral imaging method^19^. Huang *et al*. studied color feature in vegetable soybean during drying based on hyperspectral imaging^20^. All of these studies showed the feasibility of hyperspectral imaging technique for color determination.

This study investigated the determination of egg color and eggshell strength values and detection of cracked eggs using hyperspectral imaging technique. The specific objectives of this study were: (1) to detect egg color and eggshell strength values by using spectral information; (2) to select significant wavelengths for predicting color and eggshell strength values and (3) to develop a technique to classify intact and cracked eggs based on full and selected wavelengths, respectively.

## Results and Discussion

### Spectral feature

**Figure 1:**
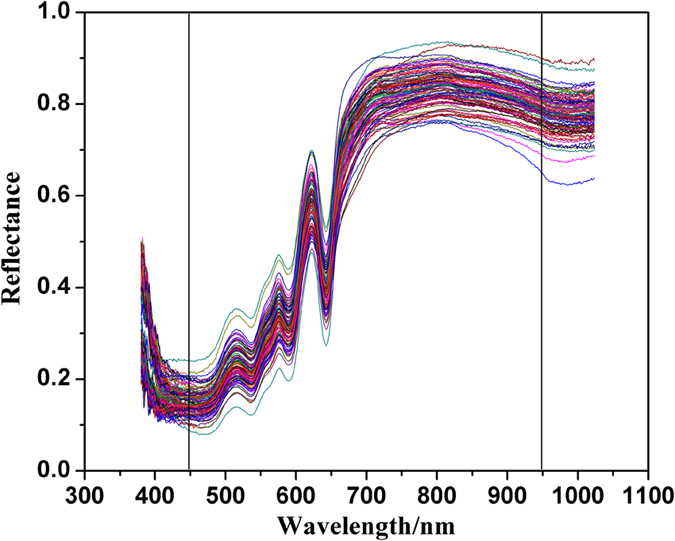
Spectral reflectance curves of samples.

Spectral reflectance curves of all chicken eggs were shown in Fig. 1. It is obviously there was some noise at the beginning of the wavelengths. The general trend of the spectral reflectance curves for different eggs were very similar. In order to classify intact and cracked eggs quantitatively, the classification model should be considered using the information from spectral curves. In a previous study, wavelengths between 570 and 750 nm were used to detect egg freshness^21^. In order to reduce the noise influence, wavelengths from 450 to 950 nm were used in this study.

### Color and eggshell strength values

**Figure 2:**
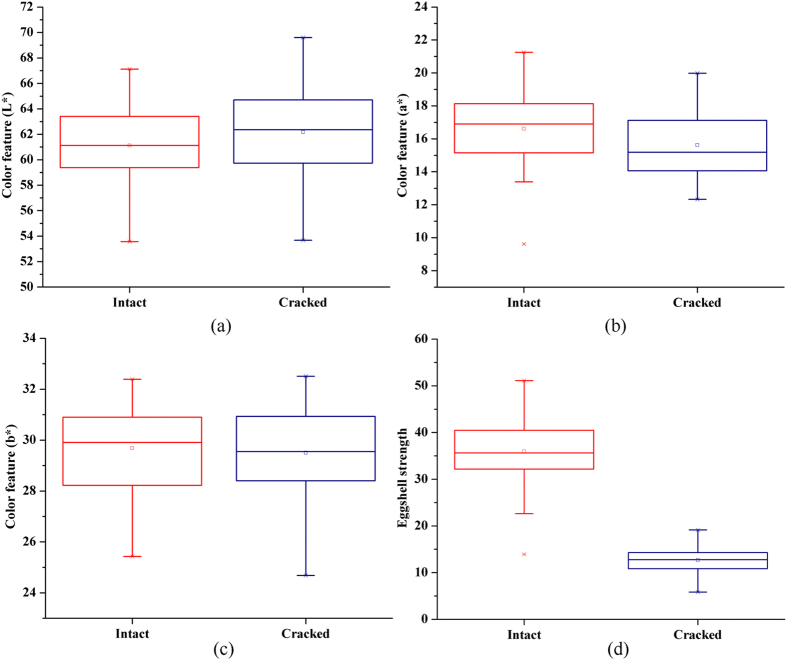
Distribution of color (L*, a* and b*) and eggshell strength values. (**a**) L*, (**b**) a*, (**c**) b* and (**d**) eggshell strength.

The minimum, maximum, mean and standard deviation of L*, a* and b* values for cracked and intact eggs can be found in Fig. 2(a–c). In this figure, the L*, a* and b* values for both intact and cracked eggs were very similar. This is because the eggs which were used to create crack were randomly selected from the total eggs. However, for each sample, it has a different and specific color feature. The eggshell strength values of intact eggs were higher than those of cracked ones as shown in Fig. 2(d). This is because cracked eggs have more fragile eggshells, directly resulting in lower values of eggshell strength. In order to avoid bias in subset partition, all samples were arranged in an ascending order according to the *Y* variables (L*, a*, b* and eggshell strength, respectively) for each model^22^. Then one egg was selected from every three ones consecutively, resulting in the calibration set and prediction set at a ratio of 2:1. No single sample was used in both calibration and prediction sets at the same time. Thus, there were 68 samples in the calibration set and 34 ones in the prediction set. The statistical values of L*, a*, b* and eggshell strength for both sets can be seen in Table 1. For each parameter, a broad range values can be found in the two sets. Therefore, the samples in both sets can represent the range of all possible values, which contributed to develop an accurate and robust model^23^.

### PLS models based on full wavelengths

Four different partial least squares (PLS) models based on the full spectral wavelengths were developed for predicting L*, a*, b* and eggshell strength, respectively. Performance of each prediction model was evaluated according to the values of correlation coefficient of calibration (*r*_*c*_), correlation coefficient of prediction *(r*_*p*_), root mean square error of calibration (*RMSEC*) and root mean square error of prediction (*RMSEP*). The results were shown in Table 2. Each model obtained a good result with high values of *r*_*c*_ and *r*_*p*_ and low values of *RMSEC* and *RMSEP*. The values of *r*_*p*_ were 0.788 for L*, 0.810 for a*, 0.766 for b* and 0.835 for eggshell strength, respectively. The results proved that spectral information could be used to determine color and eggshell strength values for chicken eggs.

### Significant wavelengths

**Figure 3:**
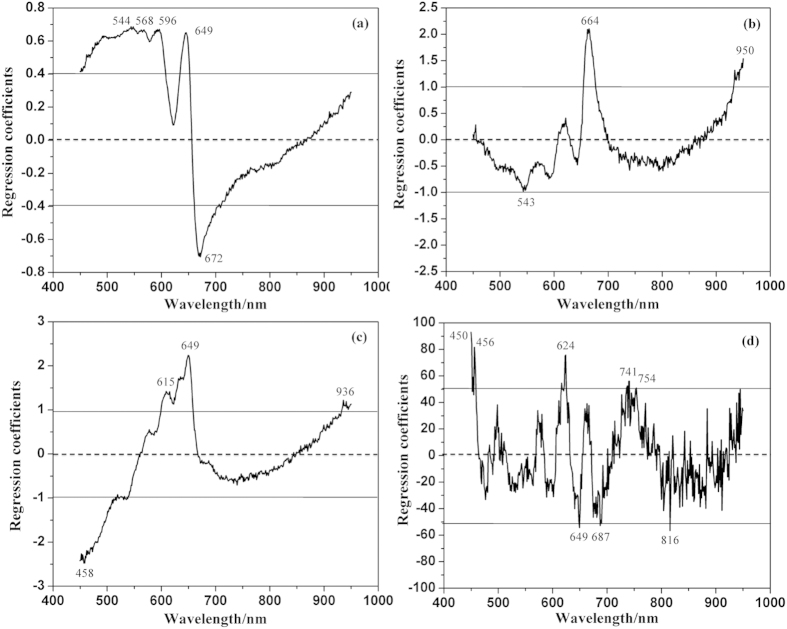
Effective wavelengths selected by RC method. The horizontal lines show the upper and lower cutoff threshold values. (**a**) L*, (**b**) a*, (**c**) b* and (**d**) eggshell strength.

In this study, regression coefficient (RC) method was used to select the effective wavelengths. The size of the coefficients gives an indication of which wavelengths were important for predicting *Y* values. It can be seen in Fig. 3(a–d) that some peaks and valleys with high absolute values were identified as the optimal wavelengths. The horizontal lines showed the upper and lower cutoff threshold values. As a result, five wavelengths were selected for L* (544, 568, 596, 649 and 672 nm), three ones for a* (543, 664 and 950 nm), four ones for b* (458, 615, 649 and 936 nm) and eight ones for eggshell strength (450, 456, 624, 649, 687, 741, 754 and 816 nm). The numbers of these selected wavelengths only took up 1.26%, 0.76%, 1.01% and 2.02% of that of the full spectral wavebands, respectively. They were then used to replace the full wavelengths for predicting L*, a*, b* and eggshell strength values.

### Prediction results based on selected wavelengths

Based on the selected wavelengths, four different RC-PLS models were established for L*, a*, b* and eggshell strength, respectively. Each model obtained a good result with high values of *r*_*c*_ and *r*_*p*_ and low values of *RMSEC* and *RMSEP* as can be seen in Table 3. Though the results based on selected wavelengths did not change too much compared with the corresponding values based on full spectral wavelengths, the number of input variables was fewer. The fewer input variables will be helpful to develop a multispectral imaging detection system. Also, it can be found the *r*_*p*_ values for b* and eggshell strength in RC-PLS models were even a little higher than those in PLS models. Thus, it proved the selected wavelengths can be equal to or even more efficient than full spectral wavelengths^24^. This may because the whole wavelengths contained too much redundant information that directly affected the prediction performance. It demonstrated that RC method could be used to identify the useful wavelengths and abandon the uninformative ones.

### Classification based on spectral information

**Figure 4:**
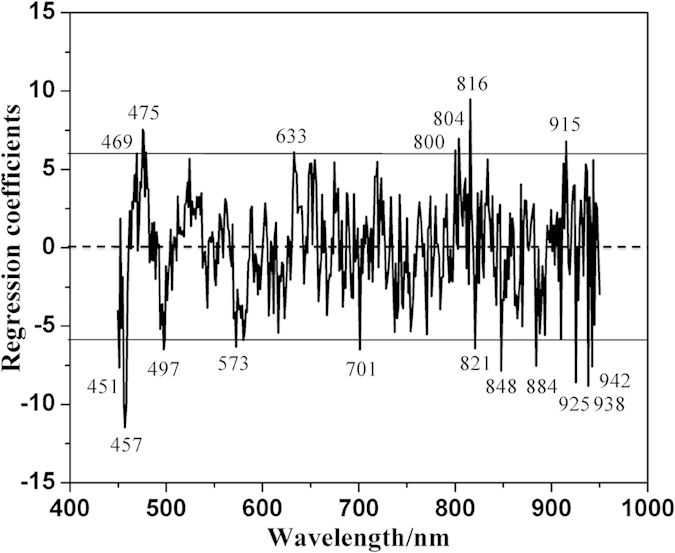
Effective wavelengths selected by RC method for classification. The horizontal lines show the upper and lower cutoff threshold values.

This study was then carried out to classify intact and cracked chicken eggs based on spectral information. Partial least squares-discriminant analysis (PLS-DA) model was firstly established to identify cracked samples. The threshold was set as ±0.3. RC method was also carried out to select the useful wavelengths for the classification model, and eighteen wavelengths (451, 457, 469, 475, 497, 573, 633, 701, 800, 804, 816, 821, 848, 884, 915, 925, 938 and 942 nm) were identified (shown in Fig. 4). They were then used to substitute the whole spectral wavelengths for building RC-PLS-DA model. The results of both PLS-DA and RC-PLS-DA models were shown in Table 4. The PLS-DA model performed excellently with the total classification accuracy (CA) of 100% in the calibration set and 97.06% in the prediction set. It also obtained a good result with the total CA of 95.59% in the calibration set and 88.24% in the prediction set for RC-PLS-DA model. Though RC-PLS-DA model performed a little worse compared with PLS-DA model, the result was acceptable. Also, the number of the input variables decreased largely, which only accounted for 4.55% of the whole wavebands. The results demonstrated that spectral reflectance information extracted from the hyperspectral images could be used to detect cracked chicken eggs effectively. Based on hyperspectral imaging technique, intact and cracked eggs can be classified effectively and non-destructively. A multispectral imaging detection system can be developed for identifying cracked eggs, which makes the detection on-line. It not only saves the cost but also speeds up the detection efficiency. When the cracked eggs are identified and taken out, there is no contamination for intact eggs, which can extend the eggs’ shelf lives and also increase the profit.

## Conclusions

The results showed that hyperspectral imaging technique could be used as an accurate and non-invasive method to predict color and eggshell strength values and detect cracked eggs. PLS models were effective for the prediction of color and eggshell strength parameters, and PLS-DA models performed excellently for the identification of cracked eggs. The numbers of the selected wavelengths for L*, a*, b*, eggshell strength features and cracked samples classification only accounted for 1.26%, 0.76%, 1.01% , 2.02% and 4.55% of that of the whole wavebands. In full wavelengths-based models (PLS), the *r*_*p*_ values for each feature were 0.788, 0.810, 0.766 and 0.835, respectively. The overall classification result was 97.06% in PLS-DA model. While the prediction results were 0.771, 0.806, 0.767 and 0.841 for the four features using RC-PLS models, and the CA was 88.24% in RC-PLS-DA model. The results based on the selected wavelengths were quite similar with those acquired by the full spectral wavelengths, which was consisted with two previous studies^13^,^25^. The wavelengths obtained in this study were useful for developing a multispectral imaging system in egg industry. In further studies, more samples need to be used for building more accurate and robust models. Also, other wavelength selection methods should be studied.

## Materials and Methods

### Samples

**Figure 5:**
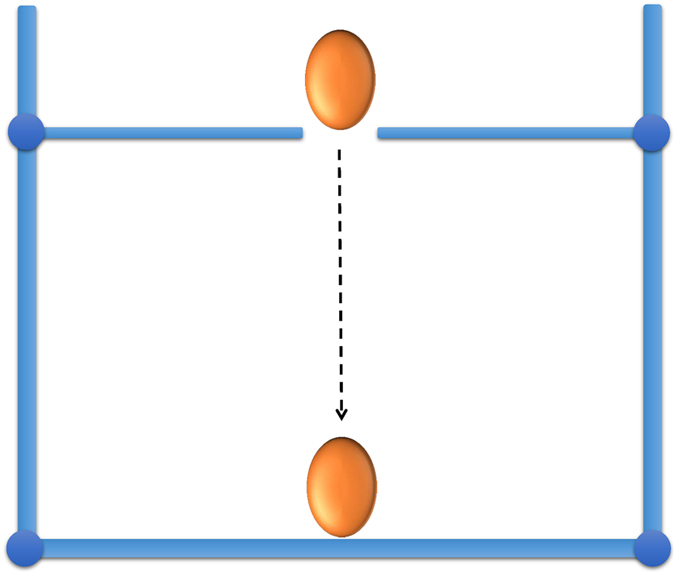
Structure diagram of the hitting device.

The eggs (fresh local eggs,) were purchased from the supermarket in China. The contamination on the surface were cleaned and then kept in the refrigerator at 4 °C. In our daily lives, most of the cracked eggs are generated in collecting, storage, transportation, as they are easily broken when contacting with other hard objectives. Thus, in order to imitate the cracked eggs generated in the real daily life, egg cracks were created by slightly hitting the experiment desk in this study. The structure diagram of the hitting device can be seen in Fig. 5. Finally, fifty-one intact and fifty-one cracked eggs were obtained for study.

### Hyperspectral imaging system and operation platform

**Figure 6:**
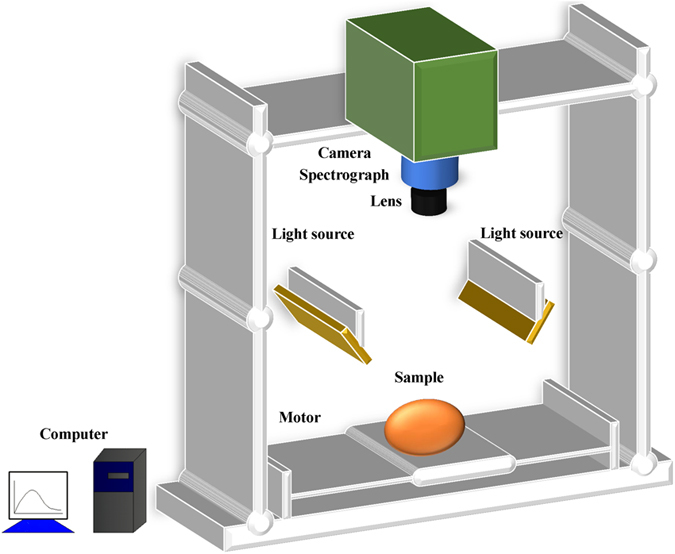
Schematic diagram of the hyperspectral imaging system.

A laboratory hyperspectral imaging system, which covers the spectral region from 380 to 1023 nm, was used in this study. The schematic diagram of the hyperspectral imaging system can be seen in Fig. 6. It consists of an imaging spectrograph (V10E, Specim, Finland), a charge coupled device (CCD) camera (C8484-05, Hamamatsu City, Japan), a lens (OLE-23), two light sources (Oriel Instruments, Irvine, USA) provided by two 150W quartz tungsten halogen lamps, a conveyer and a computer. The spectral resolution is 2.8 nm, and the area CCD array detector of the camera has 672 × 512 (spatial × spectral) pixels. All samples were scanned by the camera line by line. The ENVI 4.7 (Research System Inc., Boulder, Co., USA), MATLAB R2009a (The Math Works Inc., Natick, MA, USA) and Unscrambler V9.7 (CAMO Process AS, Oslo, Norway) software were used in this study.

### Image acquisition and correction

Before images acquisition, the exposure time, moving speed and vertical distance between the lens and samples should be adjusted in order to obtain the hyperspectral image without distortion and overexposure^26^. Finally, the exposure time was set as 0.13 s, the moving speed was 2.1 mm/s, and the vertical distance between the lens and sample was 40.2 cm. Then a white Teflon board (CAL-tile 200, 200 mm × 25 mm × 10 mm) with the reflectance of about 99% was scanned firstly, and a dark image with the reflectance of about 0% was acquired by covering the camera lens with its cap and turning off the light. Each egg was placed on the moving conveyor to be scanned line by line. For cracked samples, the cracks were oriented to the hyperspectral imaging camera. Finally, the hyperspectral images with the spectral wavelengths from 380 to 1023 nm were acquired. Each hyperspectral image had 672 pixels in the spatial dimension and 512 bands in the spectral dimension. Once the raw hyperspectral image was generated, it should be corrected based on the dark and white images according to equation (1).


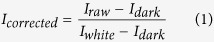


where *I*_*corrected*_ is the corrected hyperspectral image, *I*_*raw*_ is the raw hyperspectral image, *I*_*dark*_ is the dark image, and *I*_*white*_ is the white image.

### Color and eggshell strength measurement

The three color values (L*, a* and b*) were measured by the colorimeter (Konica Minolta, CR-400, Japan) with a standard C illuminant. Before color acquisition, the colorimeter should be calibrated by a standard white calibration plate. The colorimeter should totally cover the detection area of the sample, otherwise, color features of other objectives around the sample might be acquired, which made the result incorrect. The CIELAB color scale, which is a three dimensional cube color space, can represent the three color parameters (L*, a* and b*) precisely^27^. Eggshell strength was determined by the egg shell force gauge (ESFG-1, Nanjing Wanma Uitrasonic Motors Co., Ltd, China). When the egg was put on the plate, a probe would move down. It didn’t stop until the egg was broken. Then, the value shown on the screen was the eggshell strength. The unit of eggshell strength is N.

### Models and evaluation index

PLS models were built to predict the color and eggshell strength values in this study. This method is very effective in predicting collinear variables, and has been used in many previous studies^28^,^29^,^30^. The prediction result is acquired by extracting a set of orthogonal factors, which contain most of the useful information^31^. PLS method can also be used for discrimination analysis in the form of PLS-DA. This method can explain differences between overall class properties, thus, the interpretation becomes more complicated with the class number increasing^32^. PLS-DA models were established for identifying the cracked eggs in this study. Both PLS and PLS-DA models were calculated using Unscrambler V9.7 software. Performance of prediction models were evaluated according to the values of *r*_*c*_, *r*_*p*_, *RMSEC* and *RMSEP*. Excellent prediction models should have high values of *r*_*c*_ and *r*_*p*_, low values of *RMSEC* and *RMSEP*^33^. The performance of classification model was determined by CA value, which should be between 0% and 100%. The higher the CA value, the better the classification model. The equations for *r* and *RMSE* could be defined as follows:


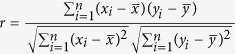



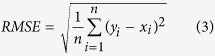


where 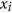 is the measurement value of the sample *i*; 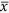 is the average value of 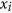; 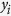 is the predicted value of the sample *i*; 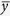 is the average value of 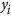; 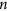 is the number of samples.

### Regression coefficient

In order to improve the prediction performance and simplify the model, effective wavelengths were then selected. These selected wavelengths aimed at identifying a small subset of spectral features to replace the full spectral wavelengths. Selected wavelengths can produce results that a better or identic to results obtained using the whole wavelengths^24^. RC method was applied to select the useful wavelengths in this study. This method is very efficient for selecting key wavelengths and has been used in many previous studies^34^,^35^. In RC algorithm, the high positive and negative peaks represent the wavelengths at these points contain the most effective information^36^. The RC algorithm was operated in Unscrambler 9.7 software.

### Experiment design

All samples were scanned by the hyperspectral imaging system firstly. Then the three color values (L*, a* and b*) were measured by the colorimeter, and the eggshell strength was determined by the egg shell force gauge. Spectral reflectance information was extracted from the corrected hyperspectral image and treated as the independent variable (*X* variable). PLS models were established to predict the three color parameters and eggshell strength value. The significant wavelengths were selected by RC method. Based on these selected wavelengths, RC-PLS models were built for the prediction of color and eggshell strength values. In this study, PLS-DA and RC-PLS-DA models were then applied to detect cracked chicken eggs.

## Additional Information

**How to cite this article**: Xie, C. and He, Y. External characteristic determination of eggs and cracked eggs identification using spectral signature. *Sci. Rep*. **6**, 21130; doi: 10.1038/srep21130 (2016).

## Figures and Tables

**Table 1:** Color (L*, a* and b*) and eggshell strength values in calibration and prediction sets.

Statistics	Calibration				Prediction			
	L*	a*	b*	eggshell strength /N	L*	a*	b*	eggshell strength /N
Minimum	53.56	9.61	25.43	5.84	56.02	12.94	24.68	8.08
Maximum	69.60	21.25	32.51	50.5	67.12	19.91	32.39	51.13
Mean	61.89	16.10	29.60	24.02	61.16	16.15	29.58	24.88
S.D.^a^	3.53	2.19	1.63	12.91	3.11	2.02	1.74	13.23

*a: Standard Deviation*.

**Table 2:** Prediction results of PLS models for color (L*, a* and b*) and eggshell strength values.

Parameters	Calibration				Prediction			
	*r*_*c*_	*RMSEC*	slope	offset	*r*_*p*_	*RMSEP*	slope	offset
L*	0.817	2.019	0.667	20.608	0.788	1.899	0.617	23.637
a*	0.834	1.199	0.695	4.912	0.810	1.224	0.519	7.903
b*	0.816	0.936	0.665	9.911	0.766	1.115	0.519	14.311
eggshell strength	0.869	6.347	0.755	5.899	0.835	7.356	0.800	5.452

**Table 3:** Prediction results of RC-PLS models for color (L*, a* and b*) and eggshell strength values.

Parameters	Number of variables	Calibration				Prediction			
		*r*_*c*_	*RMSEC*	slope	offset	*r*_*p*_	*RMSEP*	slope	offset
L*	5	0.813	2.038	0.661	20.996	0.771	1.967	0.577	26.101
a*	3	0.817	1.251	0.668	5.347	0.806	1.223	0.545	7.538
b*	4	0.773	1.026	0.598	11.911	0.767	1.102	0.558	13.068
eggshell strength	8	0.859	6.564	0.738	0.309	0.841	7.069	0.732	6.350

**Table 4:** Classification results of PLS-DA and RC-PLS-DA models.

Models	Type	Number of variables	Calibration			Prediction		
			No.^a^	Correct	CA/%^b^	No. ^a^	Correct	CA/% ^b^
PLS-DA	Intact	396	34	34	100	17	16	94.12
	Cracked	396	34	34	100	17	17	100
	All	396	68	68	100	34	33	97.06
RC-PLS-DA	Intact	18	34	31	91.18	17	17	100
	Cracked	18	34	34	100	17	13	76.47
	All	18	68	65	95.59	34	30	88.24

*a: Number of samples; b: Classification accuracy*.
